# Identification and validation of m^6^A RNA regulatory network in pulpitis

**DOI:** 10.1186/s12903-023-03578-8

**Published:** 2023-11-17

**Authors:** Hui Xu, Guangjin Chen, Jiaying Zhou, Xukang Zhou, Pengcheng Wang, Chunhui Chen, Zhi Xu, Fengyuan Lv, Xiaofang Li

**Affiliations:** 1 Department of Stomatology, Affiliated Jinhua Hospital, Zhejiang University School of Medicine, Jinhua Municipal Central Hospital, JinHua, China; 2 Department of Stomatology, Union Hospital, Tongji Medical College, Huazhong University of Science and Technology, Wuhan, 430022 China; 3 School of Stomatology, Tongji Medical College, Huazhong University of Science and Technology, Wuhan, 430030 China; 4 Hubei Province Key Laboratory of Oral and Maxillofacial Development and Regeneration, Wuhan, 430022 China; 5 College of Medicine, Huzhou University, Huzhou, 313000 China; 6 Department of Health Management Center, Affiliated Jinhua Hospital, Zhejiang University School of Medicine, Jinhua Municipal Central Hospital, JinHua, China; 7 Center of Stomatology, Tongji Hospital, Tongji Medical College, Huazhong University of Science and Technology, Wuhan, 430030 China; 8 Present Address: Hubei Province Key Laboratory of Oral and Maxillofacial Development and Regeneration, Wuhan, 430022 China

**Keywords:** Pulpitis, Gene expression, m^6^A modification, Inflammation

## Abstract

**Background:**

N6-methyladenosine (m^6^A) RNA modification regulators play an important role in many human diseases, and its abnormal expression can lead to the occurrence and development of diseases. However, their significance in pulpitis remains largely unknown. Here, we sought to identify and validate the m^6^A RNA regulatory network in pulpitis.

**Methods:**

Gene expression data for m^6^A regulators in human pulpitis and normal pulp tissues from public GEO databases were analyzed. Bioinformatics analysis including Gene ontology (GO) functional, and Kyoto encyclopedia of genes and genomes (KEGG) pathway analyses were performed by R package, and Cytoscape software was used to study the role of m^6^A miRNA-mRNA regulatory network in pulpitis. Quantitative real-time PCR (qRT-PCR) was performed to validate the expression of key m^6^A regulators in collected human pulpitis specimens.

**Results:**

Differential genes between pulpitis and normal groups were found from the GEO database, and further analysis found that there were significant differences in the m^6^A modification-related genes *ALKBH5*, *METTL14*, *METTL3*, *METTL16*, *RBM15B* and *YTHDF1*. And their interaction relationships and hub genes were determined. The hub m6A regulator targets were enriched in immune cells differentiation, glutamatergic synapse, ephrin receptor binding and osteoclast differentiation in pulpitis. Validation by qRT-PCR showed that the expression of methylases METTL14 and METTL3 was decreased, thus these two genes may play a key role in pulpitis.

**Conclusion:**

Our study identified and validated the m^6^A RNA regulatory network in pulpitis. These findings will provide valuable resource to guide the mechanistic and therapeutic analysis of the role of key m^6^A modulators in pulpitis.

## Introduction

Pulpitis is a common disease in the oral cavity with a high incidence. Pulpitis is an inflammatory disease of the dental pulp which is caused by a progressive inflammatory reaction of the pulp-dentin complex caused by deep caries, trauma, etc. [1]. There is still no new breakthrough in the treatment of pulpitis at present [2]. The treatment for pulpitis is still root canal therapy to remove the pulp tissue. However, after the pulp tissue is removed from the affected tooth, there is no nutritional supply, and the brittleness increases, resulting in a decrease in the service life of the tooth. Pulpitis, as an inflammatory disease, is closely related to epigenetic modifications [3]. Research has shown that DNA methylation and RNA modification are involved in the occurrence and development of this disease [4, 5]. However, the mechanisms involved have not yet been fully elucidated, and the urgent search for the pathogenesis of pulpitis and new therapeutic targets is expected to bring good news to patients with pulpitis.

RNA in organisms can be post transcriptionally modified by more than 100 different chemical modifications, among which N6-methyladenosine (m^6^A) has been identified as the most abundant internal modification in eukaryotic mRNA [6]. m^6^ A is associated with many diseases such as cancer [7], and viral infection and inflammation are also diseases affected by m^6^ A [8, 9]. m^6^A modification is reversible and has become one of the current research hotspots. It is catalyzed by “writers” (METTL3, METTL14 and WTAP) to form a methyltransferase complex, which adds a methyl group to the N6 site of adenine in the RRACU sequence motif (R means G or A), while demethylation the enzymes ALKBH5 and FTO reverse this process, acting as an “eraser” [10]. When modified with m^6^A, transcripts can be recognized by “readers” (such as YTHDF1-3, YTHDC1 and IGF2BPs) to perform corresponding regulatory functions [11]. However, the role of m6A modification in the development of pulpitis disease remains unknown.

Bioinformatics studies on data from microarrays have emerged as promising and efficient tools for screening disease-related genes [12, 13]. This study used public datasets and bioinformatics analysis to identify the m^6^A RNA regulatory network in pulpitis. And the corresponding tissue samples were collected to further verify the expression of key related m^6^A regulators in pulpitis. This study can provide clues for further research on the occurrence and development mechanism of clinical pulpitis disease, which is crucial for identifying new targets for early detection and treatment of pulpitis.

## Methods

### Human samples

Human pulp tissue samples were obtained from patient dental specimens aged 18–35 years, all from completely extracted third molars (12 women and 8 men; age 25.65 ± 4.9 years). Pulpitis samples were obtained from 10 patients (6 women and 4 men; age 26.5 ± 5.2 years) with teeth diagnosed as irreversible pulpitis with a previous history of spontaneous pain and caused by cold and/or heat testing of persistent pain. These teeth had clinical and radiographic evidence of deep carious lesions extending into the pulp cavity. Control samples were obtained from 10 patients (6 women and 4 men; age 24.8 ± 4.8 years) with fully erupted teeth, intact crowns, and surrounded by healthy periodontal tissue, from orthodontic indications extracted third molar. The patients underwent tooth extraction after obtaining informed consent. This study is compliant with the Declaration of Helsinki. This study protocol was approved by the Ethics Committee of Jinhua Hospital Affiliated to Zhejiang University (No.S96; 16/05/2022).

### Data collection and differentially analysis

The gene expression profile of human pulpitis was downloaded from GSE77459 through Gene Expression Omnibus (GEO) database (https://www.ncbi.nlm.nih.gov/geo/), which includes 6 normal dental pulp tissues and 6 inflammed pulp tissues. “Limma” R package were used to screen the differentially expressed genes (DEGs) and LogFC > 0.7 and P < 0.05 were set as the criterion. The volcano plot was visualized by “ggplot2” R package. The m^6^A regulators including “writers”: METTL3, METTL14, WTAP, ZC3H13, METTL16, ZCCHC4, RBM15 and RBM15B, “erasers”: FTO and ALKBH5, and “readers”: HNRNPA2B1, HNRNPC, IGF2BP1, IGF2BP2, IGF2BP3, YTHDC1, YTHDC2, YTHDF1, YTHDF2 and YTHDF3. The targets of differentially expressed m^6^A genes were acquired form m6A2Target database (http://m6a2target.canceromics.org).

### Functional enrichment analysis

We further performed the Kyoto Encyclopedia of Genes and Genomes (KEGG) [14–16] and Gene Ontology (GO) pathway enrichment analyses including biological processes (BP), cellular components (CC) and molecular functions (MF) analysis of the targets of differential m^6^A genes by “clusterProfiler”, “org.Hs.eg.db” and “GOplot” R package.

### Construction of miRNA-mRNA network of pulpitis-related m^6^A regulators

Upon the pulitis-related m^6^A regulators identified, we also predicted the potential interacted miRNA through miRwalk database (http://mirwalk.umm.uni-heidelberg.de/). Then we constructed and visualized the miRNA-mRNA network which showed the miRNAs interacting with the key m^6^A regulators in human pulpitis by Cytoscape software.

### RNA isolation and real-time PCR analysis

Total RNA from dental pulp tissue was extracted using TRIzol (Vazyme, Nanjing, China). Reverse transcription reagents (Vazyme, Nanjing, China) were used for cDNA synthesis. Real-time PCR was performed on an ABI 7300 Real-Time PCR System (Applied Biosystems, Carlsbad, CA, USA) using the SYBR Green PCR protocol. Relative mRNA expression of target genes was normalized to GAPDH using the 2^−ΔΔCT^ method and expressed as the mean ± SD of the number of replicates. The primers and primer sequences used in this experiment are listed in Table 1.

**Table 1:** Primers for PCR analysis

Genes	Forward primer 5’-3’	Reverse primer 5’-3’
ALKBH5	CGGCGAAGGCTACACTTACG	CCACCAGCTTTTGGATCACCA
METTL3	TTGTCTCCAACCTTCCGTAGT	CCAGATCAGAGAGGTGGTGTAG
METTL14	GAACACAGAGCTTAAATCCCCA	TGTCAGCTAAACCTACATCCCTG
METTL16	TTCTGTCAAGGTCGGACAATG	CAGCACCACGAATGTTATGGG
RBM15B	TACACGGAGGCTACCAGTACA	GTCGTACAGCCCGTAGTAGTC
YTHDF1	ACCTGTCCAGCTATTACCCG	TGGTGAGGTATGGAATCGGAG
YTHDF3	TCAGAGTAACAGCTATCCACCA	GGTTGTCAGATATGGCATAGGCT
ZCCHC4	CCCTCACGGACCCACTCTT	GCAAGTCTAGCTCCTGACAAC

### Statistical analysis

The statistical analyses were carried out using Graphpad software. The data were manifested as means ± SD. The independent-sample t-test was applied for the comparisons between the normal and pulpitis group. P value < 0.05 was set as statistically significant.

## Results

### Identification of pulpitis-associated m^6^A regulators

**Fig. 1:**
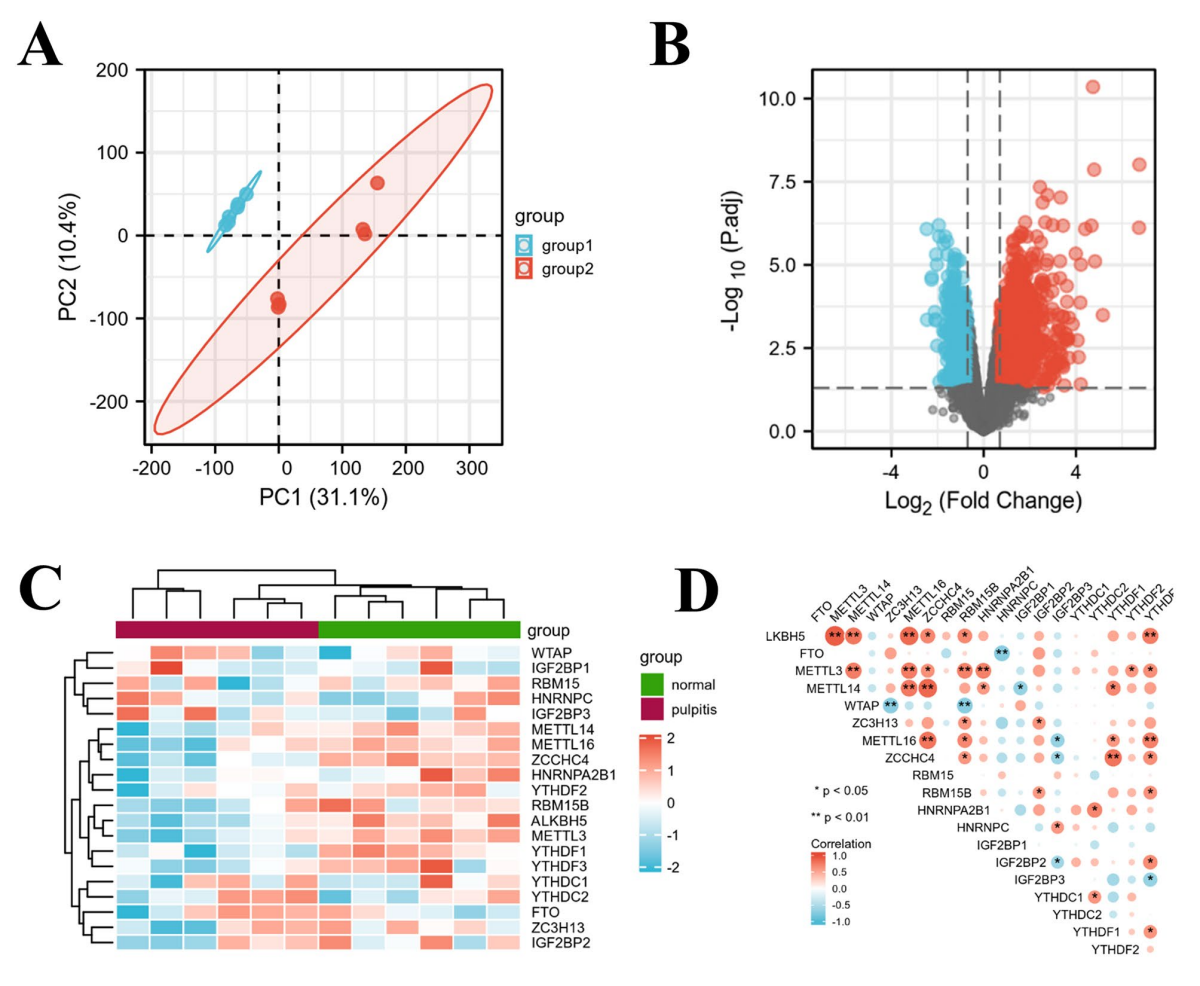
Identification of pulpitis-associated m^6^A regulators. (**A**) PCA plot: Differential gene significant grouping between normal group and pulpitis group. (**B**) Volcano plot: Differentially expressed genes between pulpitis group and normal group. Red indicates up-regulated mRNAs in pulpitis, blue indicates down-regulated mRNAs in pulpitis, and black indicates no differentially expressed mRNAs. (**C**) Heatmap of gene expression in pulpitis and normal groups. (**D**) Correlation analysis among m^6^A-related genes in pulpitis

To explore the functional m^6^A regulators in pulpitis, we preformed the differential analysis of GSE77459 dataset of pulpitis. Firstly, PCA analysis was performed and we found that the differential genes of the normal group and the pulpitis group were significantly grouped (Fig. 1A). Further differential expression analysis of genes between pulpitis tissue and normal pulp tissue was performed, as shown in the volcano plot (Fig. 1B). Then, the m^6^A-related genes were selected from these genes, including “writers”, “erasers” and “readers”, and further analysis was performed, showing the changes in m^6^A regulator gene expression between the pulpitis group and the normal group in Fig. 1C. Finally, the correlation analysis of these genes was carried out (Fig. 1D), and it was found that these genes were basically significantly correlated in pulpitis.

**Fig. 2:**
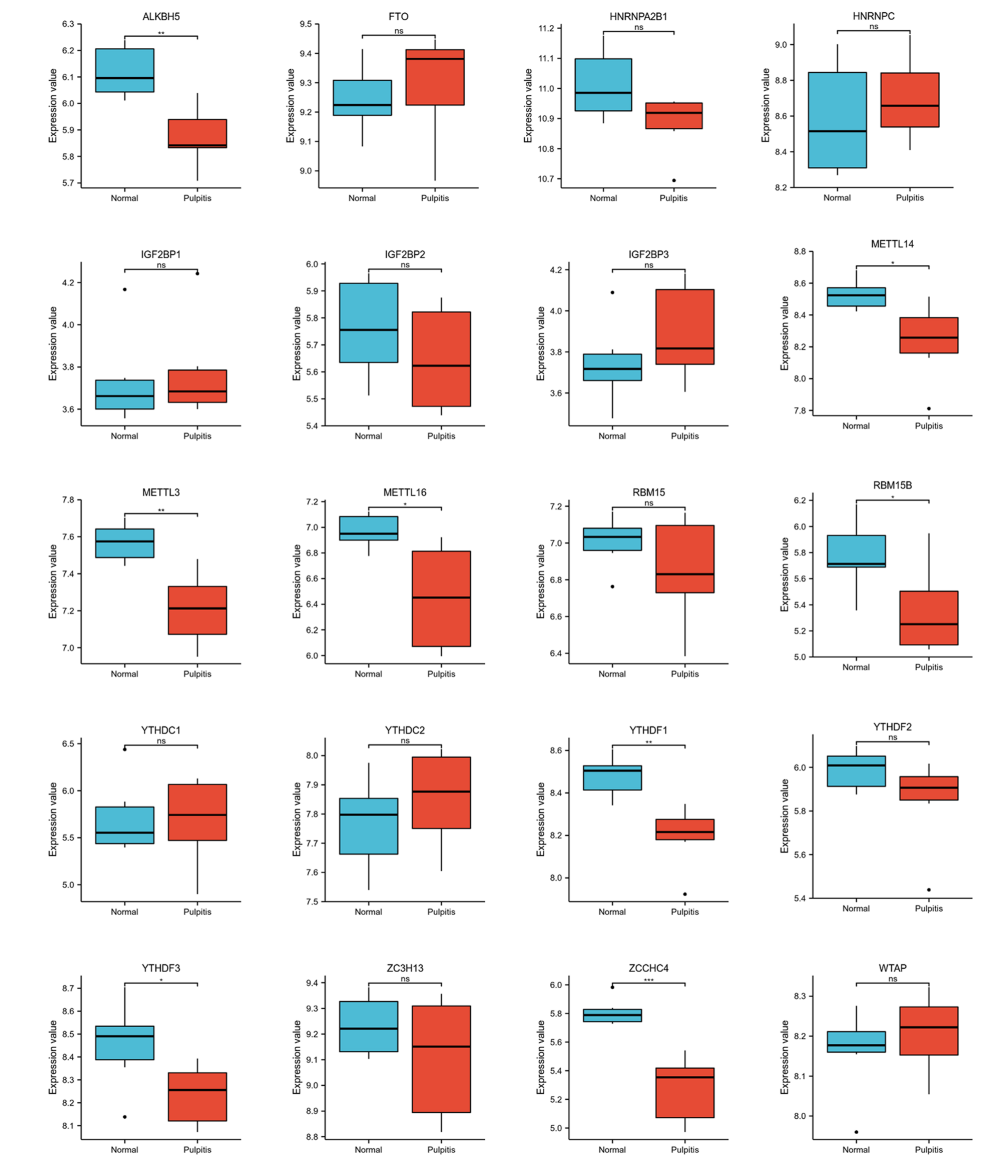
Box plots for m^6^A-related genes expression between normal group and pulpitis group. *P < 0.05, **P < 0.01, ***P < 0.001

We further compared the expression of m^6^A-related genes in the normal group and the pulpitis group and made corresponding statistical analysis (Fig. 2). The results showed that there were significant differences in the genes ALKBH5, METTL14, METTL3, METTL16, RBM15B and YTHDF1 between normal group and the pulpitis group. It indicates that these genes may play specific roles in pulpitis.

### m^6^A-related genes molecular interaction network and core genes interaction network

**Fig. 3:**
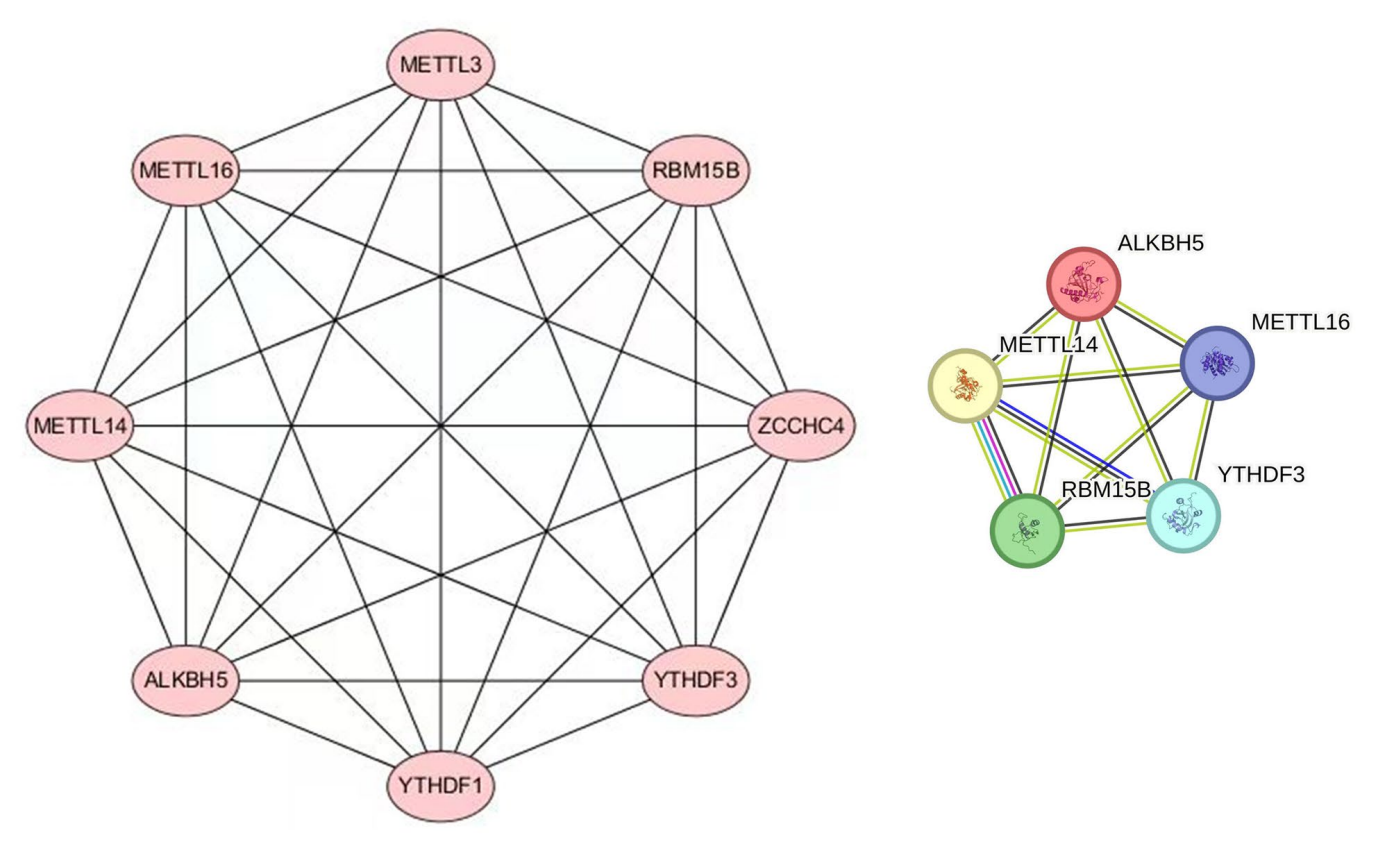
m^6^A-related genes molecular interaction network and core genes interaction network

To explore the hub m^6^A regulators in pulpitis, we further constructed the molecular interaction network of m^6^A-related genes, and Cytoscape was used to extract the interaction network of core genes (Fig. 3), which further suggested that these genes RBM15B, METTL16, METTL14, YTHDF3 and ALKBH5 may be the main regulators in pulpitis m^6^A-modified genes.

### Potential functions of genes modified by m^6^A regulators associated with pulpitis

**Fig. 4:**
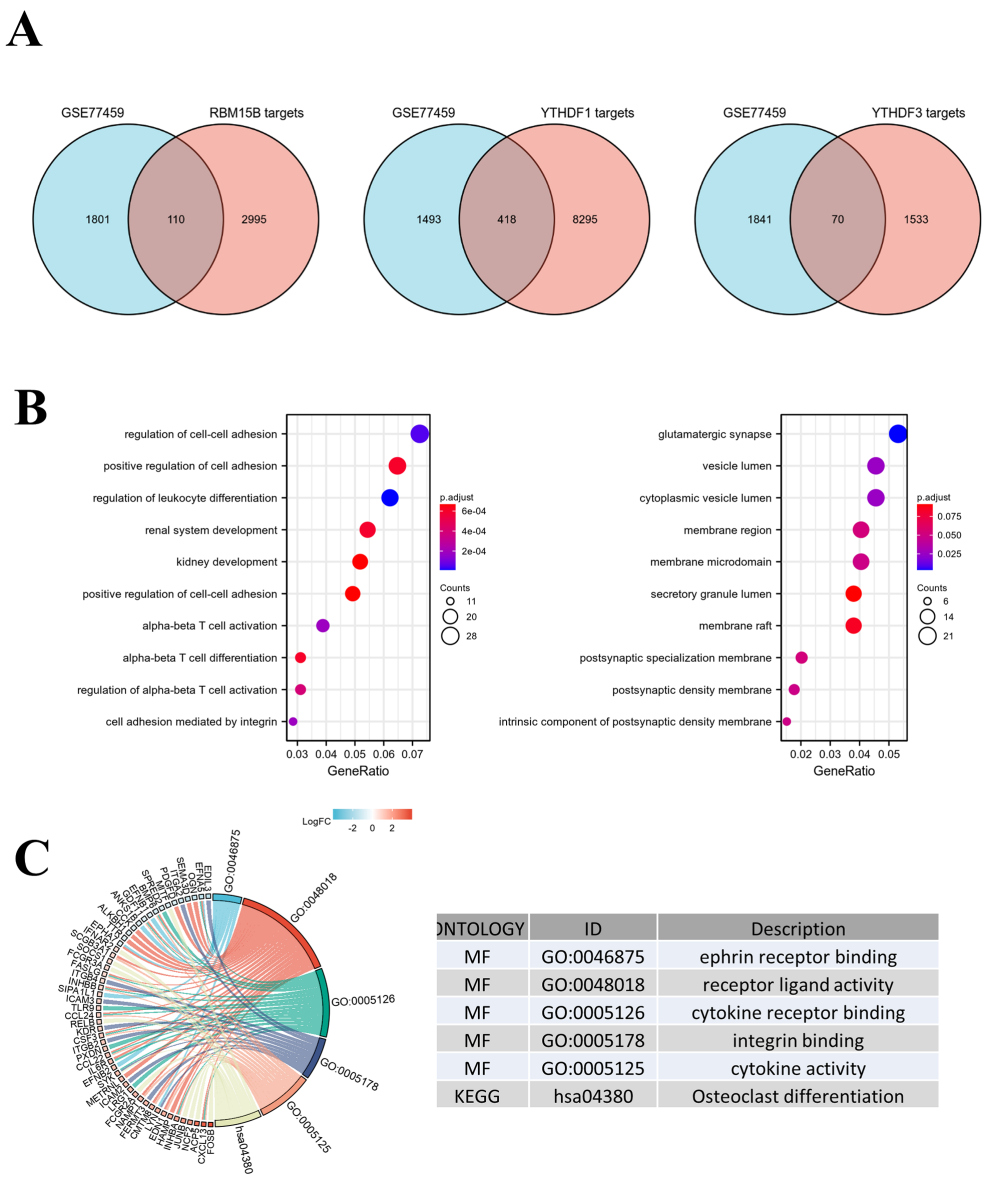
(**A**) The intersection of the downstream target molecules of the above core genes and the differential genes in GEO. (**B-C**) GO enrichment analysis and KEGG analysis

To explore the role of m^6^A regulators in pulpitis, we further analyzed the downstream target molecules of core genes with statistical significance and intersected these molecules with differential genes in GEO (Fig. 4A), suggesting that these intersected molecules are m^6^A regulated molecules in pulpitis. And the number of intersected gene between GSE77459 and RBM15B targets, GSE77459 and YTHDF1 targets and GSE77459 and YTHDF3 is 110, 418, 70, respectively. We performed GO enrichment analysis and KEGG enrichment analysis at the same time (Fig. 4B-C), and the results showed that these further screened downstream genes were mainly enriched in biological processes such as cell adhesion, T cell activation, glutamatergic synapse, ephrin receptor binding, cytokine receptors, and osteoclasts differentiation. These results suggested that m^6^A regulators in pulpitis are mainly associated the biological processes of immune cell activation, glutamatergic synapse, cytokine activity and osteoclast differentiation, which are important for pulpitis progression.

### Molecular interaction network of upstream miRNAs regulating the core m^6^A genes

**Fig. 5:**
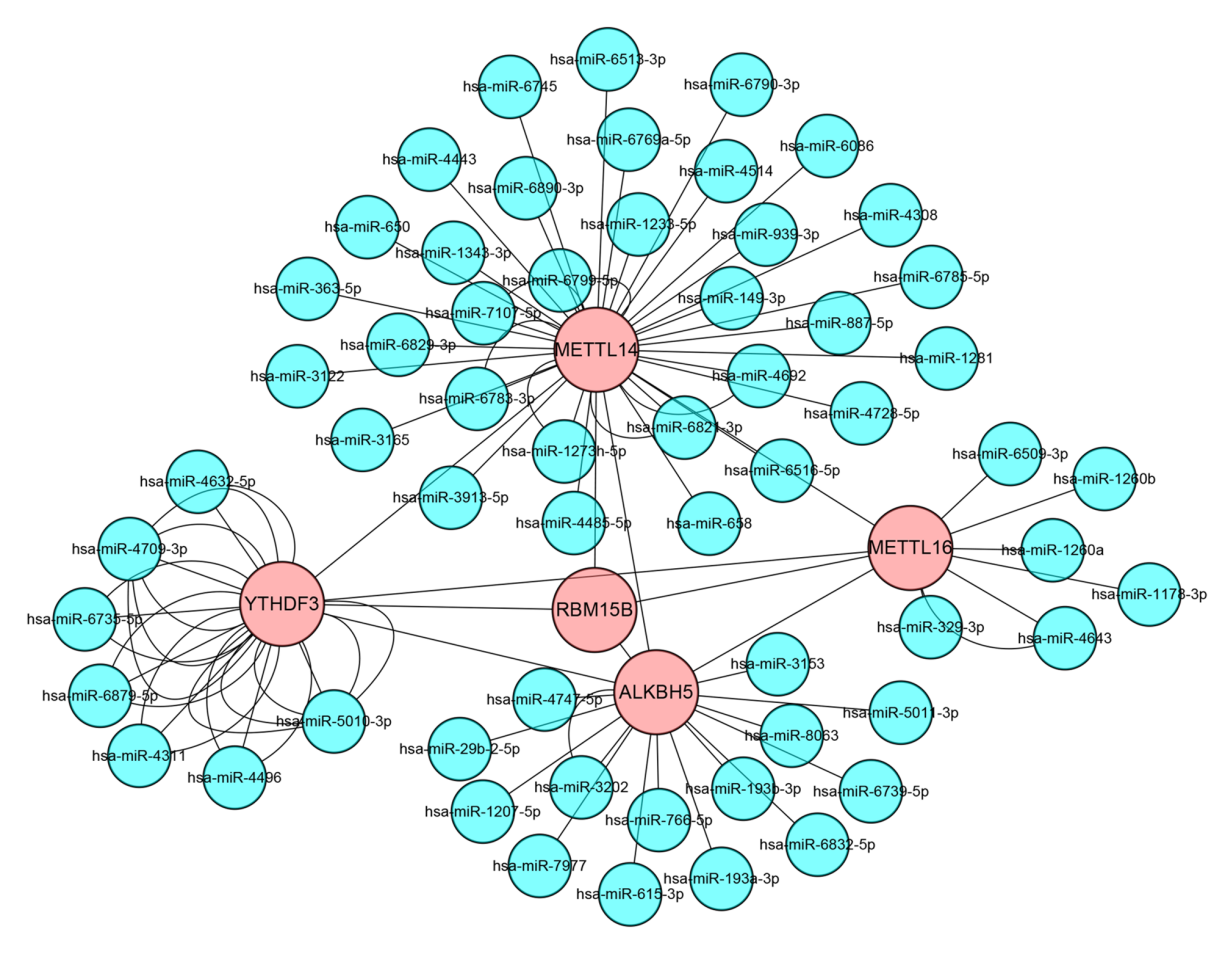
Molecular interaction network of upstream miRNAs regulating the core m^6^A genes

Pulpitis is a ubiquitous chronic inflammation in the dental pulp, and miRNAs have been found to act as promoters or inhibitors of oral inflammation [17]. To explore the potential upstream regulators of hub m^6^A genes in pulpitis, we used the miRwalkdatabase to predict the upstream miRNAs of the core m^6^A genes, including has-miR-4709-3p, has-miR-5010-3p, hsa-miR-4257, hsa-miR-550b-2-5p, has-miR-7977, has-miR-29b-2-5p, has-miR-149-3p, has-miR-363-5p, has-miR-329-3p, hsa-miR-329-3p, and made a molecular interaction network (Fig. 5).

### Validation of key m^6^A regulators in pulpitis

**Fig. 6:**
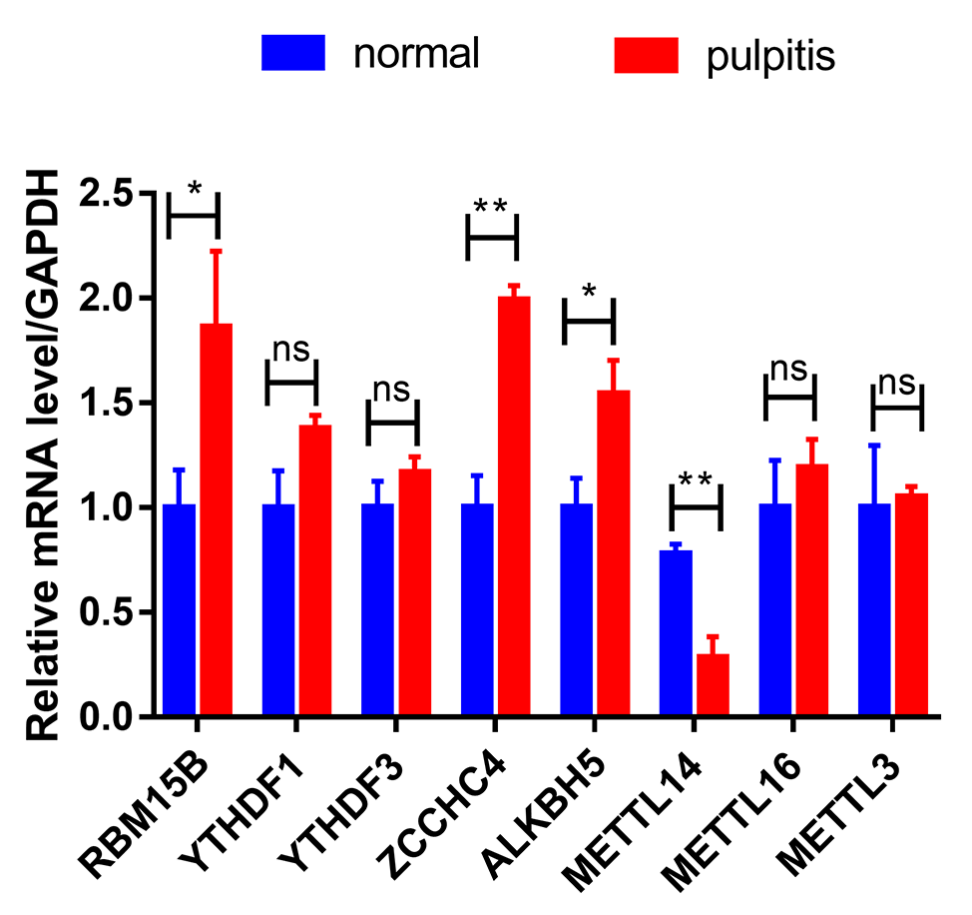
The gene expression of ALKBH5, METTL3, METTL14, METTL16, RBM15B, YTHDF1, YTHDF3 and ZCCHC4 in normal group and pulpitis tissue samples was analyzed by quantitative real-time PCR. *P < 0.05 was considered statistically significant

To further validate the key m^6^A regulators in pulpitis, the expression of ALKBH5, METTL3, METTL14, METTL16, RBM15B, YTHDF1, YTHDF3 and ZCCHC4 genes was detected by PCR on collected clinical tissue samples. METTL14 mRNA was confirmed to be significantly downregulated in pulpitis samples compared with controls (Fig. 6). However, YTHDF1, YTHDF3, METTL16 and METTL3 gene expression were not statistically different between the pulpitis group and the control group, and RBM15B, ZCCHC4 and ALKBH5 were up-regulated (Fig. 6). These results suggest that METTL14 may be a key regulator in pulpitis, further validating the differential expression of m6A-related genes between pulpitis and normal groups.

## Discussion

Pulpitis is a chronic progressive inflammatory and destructive disease that exists and occurs globally, affecting people’s quality of life to a certain extent [18]. The specific pathogenesis of the disease has not yet been elucidated [19], and it is believed to be related to a variety of factors.

In recent years, m^6^A regulators have been found to play an important role in the occurrence, development and prognosis of various human diseases [20, 21]. Studies have shown that m^6^A modification plays a role in inflammatory diseases [22], but the role of m^6^A modification in pulpitis has not been elucidated. This is the first systematic bioinformatics analysis and validation of m^6^A regulators in pulpitis disease, which can provide directions for the development mechanism of pulpitis and the targeted therapy of pulpitis.

We were the first to analyze and find the differences of m^6^A-related genes between pulpitis and normal groups, among which *ALKBH5*, *METTL14*, *METTL3*, *METTL16*, *RBM15B* and *YTHDF1* were significantly different, suggesting that these genes may be involved in the occurrence of pulpitis important role in development. These hub genes have been reported in other inflammatory diseases such as rheumatoid arthritis [23], systemic lupus erythematosus [24] and scleroderma [25], further illustrating the importance and necessity of our study of the role of m^6^A in pulpitis. We analyzed functional enrichment analysis of key m^6^A target genes and found that these genes were mainly enriched in biological processes such as cell adhesion, T cell activation, glutamatergic synapse, ephrin receptor binding, cytokine receptors, and osteoclasts differentiation. These biological processes play an important role in the occurrence and development of pulpitis. Studies have shown that cell adhesion is related to the inflammatory progression of pulpitis and affects pulp regeneration [2]. Cytokine receptors can recruit stem cells and promote the differentiation of stem cells, which can promote the regeneration of dental pulp [26]. Invasion of caries-associated bacteria into dentin tissue resulted in enhanced osteoclastogenesis and inhibition of odontoblastogenesis [27], suggesting that osteoclast differentiation also plays an important role in the progression of pulpitis. These indicate that m^6^A is also very important for the progression of pulpitis. At the same time, we have made miRNAs that may regulate m^6^A, indicating that these miRNAs may also be important non-coding RNAs in the regulation of pulpitis.

In addition, the gene expression levels of *ALKBH5*, *METTL3*, *METTL14*, *METTL16*, *RBM15B*, *YTHDF1*, *YTHDF3* and *ZCCHC4* were detected in the collected human dental pulp tissue samples, and it was found that the expression of *METTL14* in pulpitis was lower than that in the control group. The result indicates that METTL14 may be particularly important in pulpitis, and can be used as a biomarker for the progress of pulpitis, which provides a new idea for future therapeutic targets and drug development of pulpitis.

## Data Availability

The datasets used and analyzed during the current study are available from the corresponding author on reasonable request.

## Abbreviations

m^6^A: N6-methyladenosine
GO: Gene ontology
KEGG: Kyoto encyclopedia of genes and genomes
qRT-PCR: Quantitative real-time PCR
BP: Biological processes
CC: Cellular components
MF: Molecular functions
